# Entry-to-practice competency expectations for health justice in Canadian physiotherapy curricula: A scoping review protocol

**DOI:** 10.12688/mep.19126.2

**Published:** 2022-08-30

**Authors:** Kimberly Aranas, Lina Al-Habyan, Narmeen Akhtar, Isabel Ng, Haleema Noor, Mae Poirier, Jasdeep Dhir, Sarah Wojkowski

**Affiliations:** 1 School of Rehabilitation Sciences, Faculty of Health Sciences, McMaster University, Hamilton, Ontario, L8S1C7, Canada

**Keywords:** Physiotherapy, competency-based education, entry-level, health justice, social justice, health equity, healthcare disparities, social determinants of health

## Abstract

**Background**: In Canada, physiotherapists are expected to possess and demonstrate several essential competencies upon entry-to-practice. Over the past decade, knowledge and skills relating to health justice have become increasingly important for healthcare professionals. However, health justice is still an emerging topic among Canadian physiotherapy programs and current curricula may be lacking explicit content to develop knowledge, skills and behaviours related to health justice which can be used to prepare students for entry-to-practice. This paper outlines a protocol for a planned scoping review. The purpose of this scoping review will be to examine existing Canadian entry-level competencies for physiotherapy related to health justice.

**Methods:** A comprehensive literature search will be completed on four databases: OVID MEDLINE, OVID Emcare, OVID Embase, and EBSCOhost CINAHL. This scoping review will include both quantitative and qualitative methodological study designs. A grey literature search will involve advanced Google searches. Two authors will independently screen titles and abstracts to select articles for full text review. Data extraction for each selected paper will be completed independently by two authors using the proposed data extraction form. The extracted data will be presented through tables and a narrative summary that aligns with the objectives and scope of this review.

**Conclusion:** The data collected from this proposed review will identify existing competencies and gaps related to health justice in current entry-level physiotherapy curricula. This information will assist academic programs in understanding how to integrate and identify competencies and frameworks related to health justice into Canadian physiotherapy programs to ensure students are better prepared to provide culturally competent and inclusive care and promote health justice in practice.

## Introduction

### Power, Privilege and Health Justice

Health injustice is attributed to systemic health inequity, or the unfair health differences between a population and its groups, based on social, economic, demographic and geographic factors ^1, 2^. In contrast, health justice includes the optimization of health of all people, including those of low-income, minority, or racialized groups, amongst others, and requires the protection from discrimination and unfair exclusion of health services ^3^. Therefore, health justice can only be attained when systems and structures exist to ensure all people have access to the resources and opportunities needed to obtain, maintain and sustain physical, mental and emotional well-being, regardless of previous or current health status ^1, 4– 8^. Health justice requires the development of strategies that integrate and promote parity in accessibility, quality of services and resources, as well as a voice within economic, social, cultural and political systems or structures ^1, 4– 8^. Evaluating healthcare systems and structures through a health justice lens offers a broad vantage point which considers and encompasses multiple elements, including but not limited to affordability (e.g. the costs of health services and a patient’s ability to pay within their budget ^9^); availability (e.g. the ability of health care professionals to provide their services based on the patient’s needs ^9^); and acceptability ^9^ (e.g. the compatibility between a provider’s attitudes and the attitude of the patient ^9^). Promoting health justice requires an interdisciplinary response from all fields, including health care professionals such as physiotherapists ^3^.

To fully contextualize the concept of health justice, it is important to recognize the concepts of power and privilege, and what role these concepts play in contributing to justice. *Power* refers to individuals who have opportunities to access resources and are given a voice ^10^. *Privilege* occurs when individuals have an unearned advantage due to innate characteristics such as race, ethnicity, sex, and sexuality ^11^. In contrast, oppression occurs when individuals have an unearned disadvantage based on the similar characteristics ^11^.

Nixon ^11^ describes “The Coin Model'' which illustrates the seen and unseen systemic forces that contribute to privilege and oppression, and enable unfair social structures, ultimately impacting and creating inequalities within healthcare systems ^11^. In this model, individuals with privilege are represented by the top of the coin, whereas individuals who face oppression are situated at the bottom of the coin ^11^. Individuals on the top of the coin are traditionally presented with the power and resources to implement changes, without fully understanding the privilege they hold ^11^. The Coin Model signifies the importance of having individuals on the bottom of the coin being provided with the power to address inequalities of each social structure, as they have the best understanding of the changes that need to occur ^11^. However, the Coin Model can be applied to the profession of physiotherapy to analyze the areas of privilege and power.

Historically, the physiotherapy profession has failed to provide an equal and just opportunity for individuals on the bottom of the coin to have a voice ^12, 13^. here is a clear underrepresentation of Black, Hispanic, Indigenous, and Asian physiotherapists; a 2019 report by the American Physical Therapy Association (APTA) identified 84.3% of physical therapists were white ^13^. Additionally, according to a demographic analysis of 14,135 applicant records obtained for admission cycles between 2004–2014 for Ontario physiotherapy programs, only 0.8% of the physiotherapy applicants identified as Indigenous ^14^. More recently, a 2022 cross-sectional survey among Canadian physiotherapists assessing cultural competency, cultural awareness and sensitivity, and health disparities indicated low diversity among Canadian physiotherapists and a need to improve current education regarding these health justice concepts ^15^. These statistics show areas where people in power can challenge the existing and historic inequalities within the physiotherapy profession.

However, until individuals from the bottom of the coin are included in the development, delivery, and evaluation of entry-level physiotherapy program curricula, and are more equally represented within all levels and sectors of the profession, the application of concepts related to health justice will not be achieved to the extent needed to dismantle the unjust structures within the physiotherapy profession.

### Physiotherapy Curriculum and Health Justice in Canada

In Canada, physiotherapists are expected to possess and demonstrate a number of essential competencies upon entry-to-practice ^16^. A competency is defined as, “observable ability of a health care professional that develops through stages of expertise from novice to master clinician” ^17^. As outlined in the 2017 Competency Profile for Physiotherapists in Canada *,* competencies ensure all physiotherapists have the required knowledge, skills, and attitudes for a successful transition into physiotherapy practice ^16^. Within the Competency Profile, essential competencies for entry-to-practice are organized under seven overarching domains of physiotherapy practice which include physiotherapy expertise, communication, collaboration, management, leadership, scholarship, and professionalism ^16^. An essential aspect of entry-to-practice competencies is client-centeredness and the importance of understanding and considering each client's background, as well as ensuring systems exist to protect the individual's health regardless of their socioeconomic status, origin, gender, sexual orientation, religion, or culture ^16^.

To align with the current global climate and the need for health care providers to be competent and mindful in delivering care, the current Physiotherapy Accreditation Standards (Accreditation Standards) for entry-to-practice physiotherapy programs in Canada were updated in 2020 by Physiotherapy Education Accreditation Canada (PEAC) ^5^. The standards are required to be incorporated into the respective curricula of all fifteen accredited Canadian physiotherapy programs ^5, 11^. These standards ensure students enrolled in accredited Canadian physiotherapy programs are prepared to meet licensure requirements upon graduation ^5^. In the updated 2020 Accreditation Standards, two new criteria related to social justice, health justice, human rights, diversity, and equity were added ^5^. Specifically, criteria 5.4 states “the program demonstrates a commitment to relational accountability to Indigenous peoples and their communities in curriculum content and clinical learning opportunities for students” ^18^. Moreover, criteria 5.5 states “the program demonstrates a commitment to educational and health care environments that are justice-driven and anti-oppressive that may include education related to Black and Indigenous health, critical race theory, anti-oppressive practice and evaluation of teaching materials for bias and stereotypes related to racism and other intersecting systems of inequity” ^18^. 

The implementation of these standards is imperative within Canadian physiotherapy programs to ensure the quality of care provided by and experience of graduates is reflective of societal needs. In addition, deliberate efforts must be made to educate all members of the program in justice driven concepts. This includes evaluating biases within the physiotherapy admissions process to increase the numbers of individuals in Indigenous and other aforementioned marginalized groups accepted into the program ^5^.

Despite the inclusion of concepts related to health justice, inclusivity, and cultural awareness into the Accreditation Standards, health justice is an emerging topic among Canadian physiotherapy programs, and current curricula may be lacking explicit health justice frameworks. Several other health care professions have already integrated health justice into their educational materials. For example, social justice is one of six core ethics for the social work profession in Canada and is embedded in the education of Canadian social workers as part of their scope of practice, including identification of barriers or injustices present in the community and society that may impact their patients’ social wellbeing ^19, 20^. Similarly, Canadian nursing students’ curricula feature themes related to social justice such as health equity, equitable distribution, and freedom from bias ^15^. Specifically, the entry-to-practice competencies of Canadian public health nursing considers social justice and cultural safety as a requisite in the practice of public health nurses ^21^.

Due to the importance of health justice in the context of Canadian physiotherapy education and practice, and the recent inclusion of its concepts in the Accreditation Standards, further research is required to examine how health justice has been integrated into entry-level curricula to date and to identify opportunities for improvement. Thus, the primary objective of this paper is to examine existing Canadian entry-level competencies for physiotherapy related to health justice. The secondary objective is to examine the existing entry-level competencies for physiotherapy related to health justice in countries other than Canada ^22^. The last objective is to evaluate how existing entry-level competencies related to health justice in Canadian physiotherapy practice compare to those of other countries. The overall purpose of this paper is to clearly outline explicit themes and concepts related to health justice currently being delivered, and to identify gaps and areas of improvement using a critical lens. According to critical theory, this lens includes a recognition of the fact that power is structural, and these structures are often invisible; social structures are human constructs, and often built to achieve certain ends; there is a need to focus on real inequities in society and for these inequities to persist the work of maintaining privilege and power is ongoing; and critical theory is ultimately about action for those who are victims of oppression, stigma and prejudice ^23^. Thus, the authors recognize that due to the existing and historical structures of power and privilege within the profession, current practices identified through the research for this paper, may not be indicative of what should be implemented in all Canadian physiotherapy curriculums, as the perspectives of the individuals on the bottom of the coin, those who are underrepresented and without a voice in the profession may be lacking in existing practices. As such, the results of this project will also recognize what voices and perspectives are missing to help inform next steps. 

## Methods

The reporting of the final scoping review will be guided using the PRISMA Extension for Scoping Reviews (PRISMA-ScR).

The methods of this scoping review will be based on the proposed methodological stages of a scoping review by Arksey and O’Malley, Levac *et al*., Joanna Briggs Institute, and Peters *et al.* ^24– 27^. The stages will include the following:

1. Identify the research questions using the Population, Concept and Context Framework.
2. Publish the protocol for the scoping review.
3. Carry out the search and identify relevant studies.
4. Select the studies that meet the inclusion criteria.
5. Chart the extracted data.
6. Collate, summarize and report results.

This scoping review protocol was registered on February 23rd, 2022 under: https://doi.org/10.17605/OSF.IO/R76WM

### Inclusion criteria

 Inclusion criteria were defined based on the Population, Concept and Context framework per the Joanna Briggs Institute methodology for scoping reviews ^26^. Table 1 describes the inclusion and exclusion criteria.

**Table 1.** Inclusion and exclusion criteria.

Inclusion Criteria	Exclusion Criteria
English language	Written in a language other than English
Published 2011-Present	Published before 2011
Physiotherapy students	Practicing physiotherapists
Entry-level physiotherapy competencies	
Concepts related to health justice (e.g., social justice, affordability, availability, acceptability, social determinant of health, cultural competency)	

Population: This scoping review will include articles that focus on educating physiotherapy students in entry-to-practice physiotherapy programs, physiotherapy education, and entry-to-practice competencies in Canada, as well as other countries.

Concept: This scoping review will examine how existing, entry-level competencies of Canadian physiotherapy programs relate to the concept of health justice, and how Canadian entry-level competencies for physiotherapists align with competencies related to health justice for physiotherapy students in the other countries. Concepts related to the aforementioned definition of health justice will be included.

Context: This scoping review will include articles addressing curricula for entry-to-practice physiotherapists in Canada, and other countries.

### Search strategy

The search strategy aims to identify published and unpublished literature. A primary search was conducted in Ovid MEDLINE (1946 to February 2022), with “physiotherapy” and “curriculum” searched as key terms. Terms such as, but not limited to, “health equity,” “health care disparities,” and “social determinants of health” were used to represent the concept of “health justice.” Terms derived from the titles and abstracts of relevant studies were integrated to build a comprehensive search strategy. The full search strategy for Ovid MEDLINE has been included in Table 2. Two librarians, SU and NB, within the Faculty of Health Sciences at McMaster University, Hamilton, Ontario were consulted to refine and improve the comprehensiveness of the search.

**Table 2.** Sample search strategy. **Ovid MEDLINE** Search conducted February 2022

Search	Query	Records Retrieved
#1	physical therapists.mp. or exp Physical Therapists/ or physical therapy specialty.mp. or exp Physical Therapy Specialty/ or exp Physical Therapy Modalities/ or physical therap*.mp. or physiotherap*.mp.	202,521
#2	competency based education.mp. or exp Competency-Based Education/ or curriculum.mp. or exp Curriculum/ or curricul*.mp. or competenc*.mp. or entry-level*.mp. or clinical competence.mp. or exp Clinical Competence/ or exp Education/	951,996
#3	health equity.mp. or exp Health Equity/ or health care disparities.mp. or exp Healthcare Disparities/ or health status disparities.mp. or exp Health Status Disparities/ or social determinants of health. mp. or exp "Social Determinants of Health"/ or health services accessibility.mp. or exp Health Services Accessibility/ or exp "Delivery of Health Care"/ or culturally competent care.mp. or exp Culturally Competent Care/ or universal healthcare.mp. or exp Universal Health Care/ or patient centered care.mp. or exp Patient- Centered Care/ or exp Social Justice/ or health justice.mp. or equity.mp. or health inequit*.mp. or universal health*.mp. or (health* AND (accessibility.mp. or affordability.mp. or acceptability.mp. or availability.mp.))	1,294,805
#4	#1 and #2 and #3	3378
#5	Limit #4 to (yr=“2011-Current”) and (English)	1731

In addition to Ovid MEDLINE, EBSCOhost CINAHL, Ovid Embase, and Ovid Emcare will be searched. The search strategy and the specific subject headings and keywords used will be adapted for each database used in the search. Additionally, a review of the reference lists of included articles will be undertaken to identify articles not captured in the initial searches. Given the novelty of the topic, a grey literature search will be performed to find relevant information not captured in the existing published literature. To be inclusive of all available resources, the advanced Google searches will not be limited to any country but will limited to the first five pages of results.

Only articles published in English will be included in this scoping review. Studies published in the year 2011 and onwards will be included in this scoping review. This limit was selected because literature demonstrates that there was greater dialogue regarding the integration of health justice related competencies into physiotherapy program curricula beginning in 2011 ^28^.

### Study selection

After completing the search strategy in Ovid MEDLINE, EBSCOhost CINAHL, Ovid Embase, and Ovid Emcare, all articles will be imported into a reference management software, Mendeley, and then uploaded into a review management software, Covidence ( *note:* a freely available alternate software is rayyan), to remove duplicates. Furthermore, any duplicates identified during abstract screening, full text screening, and data extraction will also be removed. Titles and abstracts will be screened and reviewed by two independent reviewers, with partners agreed upon in advance by members of our research team, to ensure accuracy. Titles and abstracts that do not explicitly address physiotherapy and physiotherapy competencies will be excluded. Articles selected for full text review after abstract and title screening will be retrieved and reviewed by pairs of reviewers for inclusion and to determine their eligibility. A full report of included and excluded articles will be addressed in a PRISMA flow chart, outlining the reason for exclusion and inclusion in our final scoping review. Conflicts related to inclusion at either the title and abstract stage or full text stage will be discussed amongst the pair of reviewers to come to an agreement. Finally, disagreements will be resolved by discussion between the two reviewers or by consulting with a third reviewer.

### Data extraction

Four independent reviewers will extract data from the included articles using the process outlined in the Joanna Briggs Institute Reviewers’ Manual ^20, 29^. A data extraction tool was created by the four reviewers and will be used during the data extraction process. A draft of the form can be found in Table 3. Information on authorship, article type, article purpose, inclusion criteria, population, setting, study design and methods, and key findings relevant to the Population, Concept, Context question will be extracted. The main outcomes of interest that will be collected during data extraction are the concepts related to competencies associated with health justice in entry to practice physiotherapy curriculums (i.e., competency-based education; entry level competencies; education level; health equity; accessibility; affordability; and acceptability). The key findings of each paper, as well as the country where the research completed will be additional outcomes used to describe the existing literature. As the intention of scoping reviews is to provide a descriptive overview of the literature, scoping reviews do not require a critical appraisal of individual studies; therefore, a risk of bias assessment will not be completed on the included papers ^22^.

**Table 3.** Draft data extraction tool.

Data Collection Form	
**Reviewer of Initials**	
**Date of Extraction**	
Article Identifiers	
**Title**	
**Author name (Last, first)**	
**Who are the researchers in this article:** **Title, job role, associated department**	
**Where are the researchers from?**	
**Date of Publication**	
**Article Country of Origin**	
Eligibility Criteria	
**Does this article include Physiotherapy education concepts?** ☐Yes ☐No	
**Does this article have entry-level physiotherapy concepts?** ☐Yes ☐No	
**Is this article’s text in English?** ☐Yes ☐No	
**Is this article published from 2011-present?** ☐Yes ☐No	
**Does this article include concepts of health justice?** ☐Yes ☐No	
Article Details	
**What was the purpose or objective of this article?**	
**Methods**	
**Do the researchers have affiliations with any organization(s)?**	☐ Yes, specify: ☐ No
**What was the design?**	**□**Quantitative: **□**Systematic Review/Meta-Analysis **□**Scoping Review **□**Randomized controlled trial **□**Cross-sectional study **□**Cohort study **□**Other, please specify: **□**Qualitative **□**Phenomenological **□**Ethnography **□**Grounded Theory **□**Narrative Inquiry/Analysis **□**Interpretive Description **□**Action Research **□**Opinion Papers **□**Case Study **□**Survey **□**Other, please specify: **□**Mixed Methods: **□**Please specify:
**What was the population for this study?**	**□**Student **□**Undergraduate **□**Graduate **□**Doctorate **□**Rehab background: **□**Physiotherapy **□**Occupational therapy **□**Speech & Language Pathology **□**Other, please specify **□**Faculty; Specify Department: **□**Other, please specify:
**Were tools or techniques used by the authors to collect or inform the data collection?**	□ No □ Yes, specify: Reference available? **□**Yes: **□**No
**Education Concept**	
**Which concept of education was included in this article:**	□Physiotherapy Curriculum □Rehab curriculum
**Health Justice Concept**	
**Which concept of health justice was included in this article:**	□Health equity □Health disparities □Health status disparities □Social determinants of health □Health services accessibility □Delivery of health care □Cultural competency □Social justice □Health justice □Health injustice □Health inequity □Health accessibility □Health affordability □Health acceptability □Other:
**Were there clear competencies/** **learning objectives outlined in the** ** paper?**	□Yes If Yes, what were they? □No If No, can you find them somewhere else?
**Who/How were these concepts decided?**	
**Who delivered the health justice concepts?**	
**How were the health justice concepts delivered?**	
**What was the development process for the curriculum?**	
**What is the specific content being delivered?**	
**How were the concepts/learning objectives assessed?**	
**Was power and privilege acknowledged during this process?**	□No □Yes Please specify:
**Context**	
**Which concept of education was included in this article:**	□Canada □ Non-Canadian country; Specify:
**Results**	
**What were the major results and themes of the study?**	
**Did the authors provide specific recommendations ?**	□No □Yes Please specify:
**Limitations**	
**What were the limitations of this study?**	
**Was power and privilege acknowledged during this process?**	□No □Yes Please specify:
**Key Definitions and Objectives**	
**Were there any key definitions outlined? If so, what were they?**	
**Which of the 3 objectives in our thesis does this article address?**	□To examine existing Canadian entry-level competencies for physiotherapy related to health justice □ To examine the themes of current recommendations and written resources related to health justice for entry-level physiotherapists in countries other than Canada that have taken steps to incorporate principles of health justice into their health program curricula □ To evaluate how existing entry-level competencies related to health justice in Canadian physiotherapy practice compare to those of other countries

If revisions are made to the data extraction form, they will be reported in the final scoping review. From the studies selected for inclusion, authors will be contacted if required data is missing. Reference libraries will also be screened amongst included papers. Lastly, to mitigate disagreement between reviewers during the data extraction process, a third reviewer will be consulted if discrepancies are identified.

### Data presentation

The extracted data will be synthesized to include summary tables and graphs which reflect the frequency of key concepts described in the literature; as well as the countries or locations and the populations represented in the included studies. Additionally, the research team will review the aim and key findings of each included study and to identify if any common trends exist. These findings will be reflected in the narrative of the paper, and where appropriate, included as tables or graphs.

### Study status

At the time of protocol publication, the research team has completed searches in Ovid MEDLINE, EBSCOhost CINAHL, Ovid Embase, and Ovid Emcare using the proposed search strategy. In total 5081 records were identified across all databases. After removing duplicates, 4379 studies were uploaded to Covidence. The research team has now started abstracts and title screening.

## Conclusion

This protocol details our plan to examine the current literature on existing Canadian entry-level competencies for physiotherapy related to health justice. Furthermore, it will involve searching for trends of current recommendations and practices related to health justice for entry-level physiotherapists in other countries to compare their health justice related recommendations with that of Canada’s. After completing the scoping review, we hope to publish our results and present our findings to other health care professionals. The goal of this scoping review is to identify gaps related to health justice in current entry-level physiotherapy curricula. The use of a scoping review is a strength in this study as it is an ideal study design for emerging topics in research. Additional strengths of this study include the use of PRISMA-ScR which will provide a transparent and replicable outline of the steps in our research process. While the parameters for conducting a grey literature search will be limited, including unpublished literature will ultimately help to create a more comprehensive search. This novel area of research has great potential for making significant impacts on the Canadian physiotherapy profession and curricula. The limitations of this study include only searching four databases; limiting inclusion requirements to papers published after 2011, and papers published only in English. In regard to grey literature, the search will be limited to the first three pages of results from Canada, the United States of America, Australia, and New Zealand. Furthermore, other potential limitations may arise due to a lack of resources and limited time to conduct the study.

## Acknowledgement of Privilege and Power

The authors of this study are all physiotherapy students or registered physiotherapists who have different lived experiences and cultural backgrounds that reflect their own place of power and privilege. The authors acknowledge that although perspectives of power and privilege will be reviewed in the collected data, that the field of physiotherapy itself holds privilege in areas such as gender, colonisation and disability which will influence the data available in the existing literature ^23^.

## Data availability

No data are associated with this article.
