# Supplementary material for: Entry-to-practice competency expectations for health justice in Canadian physiotherapy curricula: A scoping review protocol
Source: MedEdPublish (2016). 2022 Aug 30;12:31. Originally published 2022 Apr 28. [Version 2] doi: 10.12688/mep.19126.2 (PMC11362729; doi:10.12688/mep.19126.2)
Supplement: Peer review history

# Reviewer report: Reviewer response for version 2

**DOI:** 10.21956/mep.20638.r33459

**Published:** 2024-08-29

**Authors:** mariya jiandani

**Affiliations:** 1 Physical Therapy School & Centre, Seth G S Medical College and KEMH, Mumbai, Maharashtra, India; 2 KEM Hospital, Mumbai, India

The article outlines a protocol for a scoping review aimed at examining the current state of entry-to-practice competencies related to health justice within Canadian physiotherapy curricula. The study is positioned within the broader context of increasing recognition of health justice as a critical component of healthcare education. It aims to identify existing competencies, highlight gaps, and provide insights for integrating health justice more explicitly into physiotherapy education.

 The relevance of this study is significant, especially given the evolving healthcare education where competencies related to social justice, equity, and inclusivity are becoming increasingly important. By focusing on Canadian physiotherapy programs, this scoping review has the potential to influence curriculum development, ensuring that future physiotherapists are better equipped to address health disparities and promote equitable healthcare practices.

**Major Points:**

- The study addresses an important and emerging topic in physiotherapy education. The authors focus on the integration of health justice into the curriculum, citing its inclusion in the updated Physiotherapy Accreditation Standards as a key reason for its importance. However, while these standards provide a framework for curriculum development, they do not fully capture the unique relevance and necessity of health justice within the physiotherapy profession.
- The protocol demonstrates an accurate methodological approach, particularly with the comprehensive search strategy across multiple databases. However, whether articles from low- and middle-income countries, would be included is not clear.
- The data extraction form is well-detailed; however the study would benefit from a more critical appraisal of the literature.

**Minor Point:**

- The article's current emphasis on accreditation standards as the primary rationale for integrating health justice into physiotherapy curricula does miss the opportunity to fully articulate these broader, profession-specific reasons. By expanding on the importance of health justice from a physiotherapy perspective, the authors could strengthen their argument, making a more compelling case for its inclusion as a core competency.

Is the study design appropriate for the research question?

Yes

Is the rationale for, and objectives of, the study clearly described?

Yes

Are sufficient details of the methods provided to allow replication by others?

Yes

Are the datasets clearly presented in a useable and accessible format?

Not applicable

Reviewer Expertise:

Physiotherapy, bioethics, Education, Cardiorespiratory Physiotherapy

I confirm that I have read this submission and believe that I have an appropriate level of expertise to confirm that it is of an acceptable scientific standard.

# Reviewer report: Reviewer response for version 2

**DOI:** 10.21956/mep.20638.r33457

**Published:** 2023-06-02

**Authors:** Filip Maric

**Affiliations:** 1 UiT The Arctic University of Norway, Tromsø, Norway

Dear authors,

 Thank you for the opportunity to review this interesting study protocol and the work you are doing in the important field of health justice. Your outlined research is highly relevant to advance justice as a central concern in health and health care yet by no means an easy field of inquiry. As a result of my reading, and based on my background in both critical physiotherapy, planetary health and environmental physiotherapy, I can offer observations on issues pertaining to the way you work with your central concept of health justice, two methodological pointers, and some minor corrections. I hope these will be valuable in further refining your study plans and their subsequent implementation. I recommend making some of the smaller changes that I mention throughout my review but leave whatever you perceive as larger issues and open questions to be handled at your discretion.

**The concept of health justice**

 One of the main challenges that your work is facing is grappling with the very concept of health justice. Overall, it seems as though a preceding study could have been one exploring the many existing definitions of the concept, or the many concepts that could be understood as relating to health justice (where the latter might function as an umbrella term?), which could then inform a curricular or literature review. Setting aside this substantial turn in focus for a potential future or parallel study, I will try to connect this topic to specific places in your writing:

 Inclusion criteria, concept: Where is your ‘aforementioned definition of health justice’? Is it the statement in sentence two in paragraph 1 of the introduction? Should this maybe be additionally represented in a separate box of its own (if you feel this is your penultimate definition)? Or are you aiming at something like a broad ‘concept field’, to the understanding of which your scoping review will contribute via the use of health justice as an umbrella term? This inclination is indicated, at least, in the notion of ‘concepts related to health justice’ and, under ‘data extraction’ when you write that ‘ *The main outcomes of interest that will be collected during data extraction are **the concepts related to competencies associated with health justice** in entry to practice physiotherapy curriculums*’. You might also want to review the ‘competencies associated with health justice’ as this specification seems to add another layer of complexity, or a different set of concepts altogether. I realize that you are interested in ‘competency expectations’ but I would argue that these would have to be grounded on a specific definition or understanding of health justice, which I am not sure you have quite established yet.

 Somewhat in line with this, I would also suggest rephrasing the related point in the data extraction tool (would ‘data collection tool’ be a better term in a critical theory-informed study that is conscious of the role of social and ecological extractivism in colonialism?): ‘Which concept related to health justice was included in the article’. In line with my latter point, you might also find that you are looking for ‘concepts related to competencies associated with health justice’, which might be an entirely different set of concepts.

 To me, ‘which concept related to health justice was included in the article’, seems the pivotal question to your whole study. Which concepts do you relate to health justice? What, for example, would you do with articles discussing the use of (critical) dis/ability theory in PT education, or even just universal design (which could readily be understood as relating to health justice for otherwise abled people) or the ICF model, or LGBTQ+ issues, or (the representation of) counter/cultural understandings of health, illness and care (as an enactment of health justice). The list goes on.

 From an environmental physiotherapy or planetary health perspective, a central extension to your conception of health justice is briefly indicated in the introduction section 1, paragraph 1, with the mention of geographic factors. Should maybe environmental factors and environmental in/justice be included as a critical element of health justice in light of past and present eco-social crises? Social and environmental injustice have also always been inseparably intertwined with colonialism and affect the health of people to this day. You could also include ecological determinants of health among ‘concepts related to health justice’, maybe even certain notions of sustainability (which point to the inclusion of future generations in our considerations of justice).

 Beyond this, the common, anthropocentric definition of health justice advanced in your study protocol reflects an attitude that is extensively addressed in the literature as no longer tenable, namely, the foregrounding of human concerns (albeit largely the concerns of some humans over those of others) over concerns for other-than-human species, ecosystems, etc. The effects of this attitude are dramatically present in the currently ongoing sixth mass extinction (i.e., the exacerbating massive loss of plant, insect, and animal biodiversity), among other large- and small-scale ecological crises. In the following article, I have tried to allude to the way in which our very definition of (health) justice might have to expand ‘to do justice’ to the crises of our time in a healthcare context: Maric, F., & Nicholls, D. A. (2022). Environmental physiotherapy and the case for multispecies justice in planetary health. *Physiotherapy Theory and Practice*, *38*(13), 2295–2306. https://doi.org/10.1080/09593985.2021.1964659

 Another point in relation to your use of the concept of health justice is whether you are focussed on a potentially overly static definition of justice, as opposed to taking to justice as a kind of ongoing praxis, a constant engagement, negotiation and re/evaluation, acknowledging ongoing shifts in stakeholders, power, interests, needs, etc etc. I am not a specialist in this particular field, but I could imagine that an inquiry into justice as a praxis could yield itself to an exploration of relevant competencies (like e.g. knowledge of and skills in the application of critical theories) in a slightly different way than a more static understanding of justice (e.g. when access, availability, affordability for all are presumably achieved).  

 Intro section 2, paragraph 3-5: I appreciate your inclusion of Nixon’s article on the Coin model but wonder whether it corresponds well with the heading to the subsection under which you have grouped this. If you finish the subsection with the sentence you are finishing it with now you give the impression that your primary focus is on dismantling health injustice within the PT profession, yet, I think this is maybe just part of what you are aiming for. If you are trying to explain the Coin model, or, precisely, the concepts of power, privilege and health justice using an inward look into the PT profession, then maybe separate this out via an additional subsection heading along the lines of ‘Understanding power, privilege and health justice: the example of the PT profession’. Another option might be to move your current first paragraph under ‘Introduction’ and then rephrase the existing subsection heading as suggested here, or similar. Then make sure you round off this subsection by stating that or how this provided you with an example to illuminate the meaning of power, privilege and health justice. 

**Methodology point 1: A critical theory lens or framing**

 Intro section 2 paragraph 5: I suggest separating the last portion of this paragraph into a subsection of its own, titled something like ‘A critical theory framing’. The last sentence of the remaining first portion of the paragraph should then maybe end on ‘using a critical theory lens’ to more accurately reflect a possible lens you might adopt, which is then further explained in the other, now sectioned-off portion of the paragraph where you explain what lens or theoretical framework you will be using in your scoping review.  

 The issue that presents itself in this paragraph is the same as the one highlighted by the previous reviewer, which is the relative lack of theoretical framing of your study so far. This is also related to the question of whether or under what conditions scoping reviews are sufficiently well aligned with ‘critical theory’ to begin with, given that, as you write under ‘data extraction’, ‘the intention of scoping reviews is to provide a descriptive overview of the literature, scoping reviews do not require a critical appraisal of individual studies’, whereas the intention of critical studies is to uncover, precisely, power and in/justices in play. With some smaller rephrasing, I would argue you can still claim the latter as the exclusion of health justice topics might itself be considered an injustice. In this case, however, you would maybe not be doing purely descriptive, but somewhat critical theory-informed work, which I think is the direction you are gradually heading. 

**Methodology 2: Inclusion criteria, population**

 You mention that you will be collecting ‘articles that focus on educating physiotherapy students in entry-to-practice physiotherapy programs’, and under ‘context’ ‘articles addressing curricula for entry-to-practice physiotherapists’.

 This got me wondering what these articles might be, and how many there might really be for you to analyse. Further, I wonder whether these articles will really be the best places where relevant concepts and competencies will be reflected? Or should you be looking at curricula instead? Or maybe even further down, the teaching content within one institution? Or maybe I misunderstood and you will also be looking at curricula and relevant guidelines? it might be worth clarifying, potentially even just by changing the word 'articles' to 'literature' as a broader term.

**Minor issues**

- Search strategy, final paragraph, suggestion: ‘there was greater dialogue regarding the integration of health justice related competencies into physiotherapy program curricula in the English-language literature beginning in 2011’.
- Intro paragraph 2: privilege and oppression do not only occur at individual but also at group level, so the sentences there should likely read ‘…occurs when groups or individuals have…’ plus setting ‘dis/advantages’ in the plural as these rarely come in the singular.
- Intro paragraph 4: First word in second sentence should likely read ‘There’
- Correct reference 23: The title of the book Physiotherapy Otherwise is currently incorrect.

Best of luck with your further writing. I look forward to reading a new version of your study protocol and, eventually, your review findings and follow-up research.

Is the study design appropriate for the research question?

Partly

Is the rationale for, and objectives of, the study clearly described?

Yes

Are sufficient details of the methods provided to allow replication by others?

Yes

Are the datasets clearly presented in a useable and accessible format?

Not applicable

Reviewer Expertise:

critical physiotherapy, environmental physiotherapy, sustainable healthcare, planetary health, healthcare education

I confirm that I have read this submission and believe that I have an appropriate level of expertise to confirm that it is of an acceptable scientific standard, however I have significant reservations, as outlined above.

## References

[1] : Environmental physiotherapy and the case for multispecies justice in planetary health. *Physiother Theory Pract* .2022;38(13) : 10.1080/09593985.2021.1964659 2295-2306 10.1080/09593985.2021.1964659 34365892

# Reviewer report: Reviewer response for version 2

**DOI:** 10.21956/mep.20638.r32516

**Published:** 2022-08-31

**Authors:** Stephanie Nixon

**Affiliations:** 1 Department of Physical Therapy, University of Toronto, Toronto, ON, Canada

My gratitude to these authors for receiving my reflections so openly. I appreciate the care taken to engage with the ideas I shared for strengthening the study, and I wish this team the very best with this challenging and important undertaking. Our field needs this work and these scholars! 

 No further recommendations from me - although I remain open to connecting should this team wish to connect at any point.

Is the study design appropriate for the research question?

No

Is the rationale for, and objectives of, the study clearly described?

Yes

Are sufficient details of the methods provided to allow replication by others?

Yes

Are the datasets clearly presented in a useable and accessible format?

Not applicable

Reviewer Expertise:

anti-oppression within the health sphere

I confirm that I have read this submission and believe that I have an appropriate level of expertise to confirm that it is of an acceptable scientific standard.

# Reviewer report: Reviewer response for version 1

**DOI:** 10.21956/mep.20484.r32007

**Published:** 2022-05-25

**Authors:** Stephanie Nixon

**Affiliations:** 1 Department of Physical Therapy, University of Toronto, Toronto, ON, Canada

This is an important inquiry that stands to contribute to the critical theoretical research emerging within the field of physiotherapy (and beyond). My congratulations to the authors on tackling this fraught and challenging topic. Below I propose several ways to ensure that the assumptions underpinning this inquiry are lined up to advance transformative change as opposed to unwittingly reproducing inequity. In short, my reflection is that, technically, the scoping study plan looks solid. However, the inquiry is missing a rigorous theoretical basis in terms of understanding how power operates, which is both common and dangerous. Let me explain.

**MOST IMPORTANT DEAL-BREAKING POINT:**  Because critical theoretical work applying an anti-oppression lens within the field of PT is relatively modest, it means that we don’t necessarily have a strong tradition or set of expectations to guide us, and what ends up happening is that common sense or surface-level understandings are taken as acceptable and normal. By “critical”, I am referring to research that is concerned with power, and the ways that it gives an advantage to some people, ideas, and ways of being over others, leading to inequities. The proposed inquiry is critical by definition with its focus on health justice, which is about power. As such, it is necessary to have a theoretically rigorous starting point for the inquiry in order to avoid the very common trap of unwittingly reproducing inequities while undertaking work designed to uproot them. At present, the framing of how power operates as the basis for understanding health justice is largely underdeveloped, which sets up serious problems for this inquiry. In a nutshell, we are missing the coins (i.e., the actual intersecting systems of inequality that produce inequity and injustice) and the top of the coin (i.e., the role of people in positions of unearned advantage or privilege in upholding and reproducing these inequities through their actions, and in this case, through their fields) (please see https://bmcpublichealth.biomedcentral.com/articles/10.1186/s12889-019-7884-9) ^1^. This shortcoming is not unique to this protocol – my experience has been that this is the dominant way of thinking about equity and social justice within the health sphere broadly (i.e., we’ve been taught that this is the common sense way to think about justice). But it’s necessary to bring in the source of inequities, and our complicity in them as a field, in order to set up this inquiry in a way that can “see” the full picture. Otherwise, we’re only looking for and at the bottom of the coin – i.e., groups who are marginalized – and reproducing the faulty idea that this is the problem to be addressed. This inquiry needs an explicit framework for entering into the study that understands the problem as **intersecting systems of inequality** (by which I mean the many ‘isms’) and the complicity of people on top of those coins in upholding these unjust systems.  This is IMPERATIVE in order for you to be able to understand and locate what you find in the scoping study, vs taking what you find at face value as right and good. As such, this power analysis/framing is about us shining a light on not just how to make things more just by being outward looking, but also looking inward at our own role as a profession in upholding these systems of inequity - i.e., cleaning up our own house. My guess is that mostly what you’ll find in this review is that there is a LACK of research/guidance about the coin and top of coin, and lots about bottom of coin – this needs to be made visible.  And that’s why you need a coherent critical orientation going in – i.e., to be able to see what’s missing. Otherwise, you are setting up the inquiry to report on the bottom of the coin details found in the literature as if it’s what we SHOULD be doing, vs locating those findings in the context of a coherent anti-oppression analysis that can show what is helpful and also what is missing. This has important implications for aspects of the study protocol. E.g., from Table 1 inclusion criteria: “Concepts related to health justice (e.g., social justice, affordability, availability, acceptability, social determinant of health, cultural competency)” – what about our settler colonial roots as both a country and a profession, racism, ableism, heterosexism and lots of other isms that play out in PT which PRODUCE and MAINTAIN the inequities that health justice is concerned about? You need to actively look for these (and others aligned with a more coherent power analysis) in order to be able to state with confidence both what you find and also what you've found to be missing/invisible in your search. This is what is called for in the two new PEAC criteria. I also spell this rationale out in this 10-min video here: https://www.youtube.com/watch?v=a30a_NiT5zc&list=PLNWUsONW1NHKByYnDkqHAFpoCXcGuIGa4&index=6 and this 23-min video here: https://vimeo.com/644652449/644179039).

**POINT 2.** From this more coherent starting point, there are a number of concepts one could put under the spotlight. I was intrigued by the decision to focus on “health justice”. In my wider anti-oppression work within the health sciences, I have not found this to be a common framing. I’m not suggesting that it needs to be changed. However, I am calling for a deeper rationale for why this concept is the focus as opposed to many other aspects of anti-oppression and social justice that are relevant in the context of PT education. And reflection on what gets foregrounded by this particular focus and what gets sidelined/eclipsed.

**POINT 3.** The choice to consider only grey literature from certain regions of the world seems curious to me for a study interested in justice. There is a tendency to see ‘high-income countries’ as more advanced and holding more expertise than ‘less developed’ countries – yet some of the most important justice movements are taking place in those settings. Given the focus on colonization as part of the health justice concept (as linked to the new PEAC criteria) within this inquiry, I encourage the authors to consider the implications of privileging grey lit from countries that are the winners of colonization and foreclosing on possible contributions from other settings. Granted, language may be an issue with the focus on English texts only, but I propose that that is a pragmatic limitation of the inquiry and not a reason to prevent the possibility of learning from a wider range of settings.

**POINT 4. ** Some more minor points that may be adjusted in attending to point 1:

- Intro para 3 – ‘cultural awareness’ likely should be ‘cultural competency’ or ‘cultural safety’ is better – the assumptions about power and complicity in these different concepts are important and need to be aligned with your elevated conceptual analysis underpinning the study
- Intro para 1, line 5 – “misrepresented” – I am unclear what this might mean
- Intro - Unlinked to power analysis, but a friendly heads-up that ‘criteria’ should be the singular ‘criterion’ at two points

I've suggested an important shift above, which will hopefully be received in the spirit of collectively holding ourselves to account within PT (and healthcare broadly) as we try to do transformative change-making work toward justice. I am in this struggle with you, also learning and unlearning.  My very best wishes for your important work!

Is the study design appropriate for the research question?

No

Is the rationale for, and objectives of, the study clearly described?

Yes

Are sufficient details of the methods provided to allow replication by others?

Yes

Are the datasets clearly presented in a useable and accessible format?

Not applicable

Reviewer Expertise:

anti-oppression within the health sphere

I confirm that I have read this submission and believe that I have an appropriate level of expertise to state that I do not consider it to be of an acceptable scientific standard, for reasons outlined above.

## References

[1] : The coin model of privilege and critical allyship: implications for health. *BMC Public Health* .2019;19(1) : 10.1186/s12889-019-7884-9 1637 10.1186/s12889-019-7884-9 31805907 PMC6896777

# Response

**Published:** 2022-07-28

**Authors:** Sarah Wojkowski

**Affiliations:** McMaster University, Canada

Response to Reviewer Dr. Stephanie Nixon Re: Entry to practice competency expectations for health justice in Canadian physiotherapy curricula: A scoping review protocol  

 Thank you to the reviewer, Dr. Stephanie Nixon, PT, PhD for their review of our scoping review protocol. The review was received with great appreciation and recognition of the important feedback provided to the project team. Considering the review provided, the project team took their time to respond to the review, and to revise the submitted protocol manuscript, to ensure a more fulsome development of concepts and process in the proposed review in a manner we anticipate will address the concerns identified.  As Dr. Nixon notes, the authors recognize this topic is challenging and still emerging in the physiotherapy profession. The lack of literature that addresses anti-oppressive, and justice-driven concepts within the profession, and more specifically related to entry-to-practice curriculum development and implementation, presents an important opportunity for Canadian physiotherapy educators who will need to prepare students to lead the transformative change in the profession noted. Additionally, the main objective of this review is to identify *how* health justice topics have been integrated to date, identify gaps, and provide recommendations for *improvement* in entry-level curricula. The reviewer provided a reference to their work on the Coin Model (Nixon 2019) to encourage the research team to state the theoretically grounded starting point for the proposed review, and to identify there was a risk of upholding and reproducing inequities through the outcome of the proposed review if not addressed.  The research team appreciated this feedback, and acknowledges, a possible outcome of this scoping review may be to illustrate an imbalanced, or lack of reporting of both sides of the coin exists in the literature and existing curriculum documents. However, to the authors’ knowledge a systematic literature review, which includes academic and grey literature, related to anti-oppression and justice driven concepts in entry-level physiotherapy curricula in Canada has not been completed. Thus, while the results of this scoping review may confirm the reviewer’s impression, they may also illustrate where intentional, collaborative changes can occur in curriculum development and implementation such that underrepresented theories, voices and lived experiences might be more explicitly included in future entry-to-practice physiotherapy curricula.   Below, we have described the changes the review team has made to the proposed protocol, and where applicable provide our rationale for the inclusion of specific content or focus.

1. Missing an explicit framework
  The authors acknowledge that the first version of the protocol did not fully frame how power operates and the influence it carries in understanding health justice. We recognize much of what was alluded to in the protocol aligned with key principles of critical theory, but this was not explicitly stated as a guiding framework. However, we also acknowledge the critiques of critical theory which include an over-simplification of power relations (i.e. a binary between those who hold power and those who do not); and lacking explanations about why power persistently creates haves and have nots, and how people facilitate the process (Nichols, 2022).  As such, the protocol now references a critical theory lens, as well as Nixon’s coin model to frame how power operates, and to set the inquiry driving this review in a manner that “sees” the full picture. Revisions to the protocol now include an enhanced introduction, which will ground the reader with a discussion of the Coin Model (Nixon 2019), as well as a discussion of which voices may be under-represented in the physiotherapy profession and in the literature identified through the review process; and an enhanced data extraction tool that explicitly identifies who the authors are, identifies the developers of any curricular content delivered, identifies the deliverers of any curricular content delivered, as well as if the concepts of power, voice and privilege have been acknowledged or discussed in the included papers. This enhanced data extraction form will help the research team be intentional about also identifying what is missing from the existing body of literature.
2. Understanding and locating what is identified in the scoping review
  The authors acknowledge that an important element of this scoping review is to identify themes and concepts that are explicit but also to see beyond what is written to capture what is silent or missed in the published literature. As such, and as noted above, the data extraction tool now includes a section to identify if power and privilege have been acknowledged in the paper, who the authors of the work are, as well as who was involved in the development, delivery and /or evaluation of any curricular content presented.
3. Deeper rationale for health justice
  The review team acknowledges that the concept of health justice is not a common framing for anti-oppressive work in the health sciences. However, Borras (2020) identifies that the concept of health justice offers a broad lens that considers economic redistribution, political representation and cultural recognition, and the intersections of these concepts.  Thus, grounding this review with the concept of “health justice” provides a framework from which multiple elements can be considered and reviewed as part of the literature search, data extraction and theming processes.  These elements include but are not limited to access (not only at the point of service, but also from a lens of affordability and acceptability from the client’s perspective); funding; and quality of services. A stronger rationale for using this concept as a frame has now been provided, as well as what gets foregrounded and what might be sidelined or eclipsed in this context.
4. Updated grey literature search
  The perspective of the reviewer on the approach to the grey literature was greatly appreciated. Based on the feedback from the reviewer, the authors have revised their approach to the grey literature search to be more inclusive, and not restrictive to only a specific list of countries. However, the limitations of including English only resources will be acknowledged as part of the study findings once the study is completed.
5. Other considerations
  The other considerations identified by this review have also been addressed:

- Cultural awareness – when the word was not used specifically by the cited literature, we have revised the protocol to reflect cultural safety; thank you for identifying this important change to terminology as use of terms is changing; the team has also acknowledged the changing terminology in the review.
- Misrepresented – Thank you for asking for clarification on this concept; this term has been replaced with ‘underrepresented’ which aligns with the findings in the cited surveys
- Criteria  - this language has been updated to reflect the singular criterion at the two points

The revised protocol has been uploaded, and the authors look forward to continued discussion on the review and the concepts included, as dialogue and continued learning is an important step in the process of identifying and challenging our profession to consider how to ensure our entry to practice curricula can implement the new standards and expectations that will help to transform the profession in the future.   

 References Nixon SA.The coin model of privilege and critical allyship: implications for health. *BMC Public Health. *2019; **19** (1): 1637 PubMed Abstract | Publisher Full Text *Nichols DA. Physiotherapy Otherwise. Tuwhera Open Access Books. 2022. Available online: https://ojs.aut.ac.nz/tuwhera-open-monographs/catalog/view/8/13/60-1*
